# SARS COV-2 virus-laden droplets coughed from deep lungs: Numerical quantification in a single-path whole respiratory tract geometry

**DOI:** 10.1063/5.0040914

**Published:** 2021-02-22

**Authors:** Xiuhua April Si, Mohamed Talaat, Jinxiang Xi

**Affiliations:** 1 Department of Aerospace, Industrial, and Mechanical Engineering, California Baptist University, 8432 Magnolia Ave., Riverside, California 92504, USA; 2 Department of Biomedical Engineering, The University of Massachusetts at Lowell, 1 University Ave., Lowell, Massachusetts 01854, USA

## Abstract

When an infected person coughs, many virus-laden droplets will be exhaled out of the mouth. Droplets from deep lungs are especially infectious because the alveoli are the major sites of coronavirus replication. However, their exhalation fraction, size distribution, and exiting speeds are unclear. This study investigated the behavior and fate of respiratory droplets (0.1–4 *μ*m) during coughs in a single-path respiratory tract model extending from terminal alveoli to mouth opening. An experimentally measured cough waveform was used to control the alveolar wall motions and the flow boundary conditions at lung branches from G2 to G18. The mouth opening was modeled after the image of a coughing subject captured using a high-speed camera. A well-tested *k-ω* turbulence model and Lagrangian particle tracking algorithm were applied to simulate cough flow evolutions and droplet dynamics under four cough depths, i.e., tidal volume ratio (TVR) = 0.13, 0.20. 0.32, and 0.42. The results show that 2-*μ*m droplets have the highest exhalation fraction, regardless of cough depths. A nonlinear relationship exists between the droplet exhalation fraction and cough depth due to a complex deposition mechanism confounded by multiscale airway passages, multiregime flows, and drastic transient flow effects. The highest exhalation fraction is 1.6% at the normal cough depth (TVR = 0.32), with a mean exiting speed of 20 m/s. The finding that most exhaled droplets from deep lungs are 2 *μ*m highlights the need for more effective facemasks in blocking 2-*μ*m droplets and smaller both in infectious source control and self-protection from airborne virus-laden droplets.

## I. INTRODUCTION

COVID-19 is a highly infectious disease that is caused by the severe acute respiratory syndrome (SARS) CoV-2 virus. Even though 82% of cases are mild, the virus' ability to spread at an alarming rate puts many people at risk of developing a severe case and overruns available medical resources. To date (Dec. 13, 2020), worldwide there have been over 70.5 × 10^6^ COVID-19 cases, resulting in over 1.6 × 10^6^ deaths.^1^ The number of cases in the United States has reached over 16 × 10^6^, resulting in over 0.3 × 10^6^ deaths.^2^ More alarmingly, daily new cases and mortality still accelerate, making interventions and control in virus transmission are more urgent than ever. Studies show that the COVID-19 virus affects the human body in an aggressive manner. Once entering the cell, it controls the cell's replication and creates millions of copies of itself, exponentially increasing its population.^3^ Evidence suggests that the COVID-19 virus uses the same receptors as SARS and has three stages of infection, namely, viral replication, immune hyper-reactivity, and pulmonary damage.^4^ In the respiratory tract, the virus seeks out and invades two types of cells: the goblet cells and the ciliated cells. The goblet cells are responsible for producing mucus that lubricates and protects the lungs from pathogens and from drying out, while cilia clear debris from the lungs by directing the mucus to the pharynx for disposal via the stomach. The ciliated cells have been proven to be preferred hosts for SARS, and they are also believed to be the ideal hosts for the novel SARS CoV-2. After infected cells die, they break into debris and spread the virus into deep lungs. The immune system will recognize and attack the virus in a highly controlled manner by limiting the inflammatory responses to the infected small areas. In severe cases, however, the immune system may damage the healthy tissues leading to more cellular necrosis. An inflammatory hyper-response, or cytokine storm, can cause an increase in permeability of the alveoli and capillary vessels, resulting in fluid leaking into the lungs and accelerating the development of pneumonia.^5^ The manifestations of severe COVID-19 disease include acute respiratory distress syndrome, hyper-inflammation, acute cardiac injury, acute kidney injury, and neurological disorders.^6^ Typically, people at senior ages and with underlying health conditions are more susceptible to developing severe cases of COVID-19.

The SARS CoV-2 virus can be transmitted through airborne particles and fomites, infected surfaces, and respiratory droplets.^7^ It can enter the human body through the eyes, nose, or mouth. When a person first contracts COVID-19, the virus can incubate inside of the body for up to 2–14 days before showing any signs of infection. Once that incubation period is over, the virus causes inflammation inside the lungs, specifically in the alveoli. Research has shown that sufficient respiratory droplets can be generated for COVID-19 transmission during speaking, coughing, and sneezing, and possibly during quiet breathing. The risk of transmission depends on multiple factors such as the exposure time, virus-laden droplet concentration, and usage of preventative measures personal protective equipment, or PPE. Examples of COVID outbreak episodes after social gatherings emphasize its transmission through close contact where virus-laden droplet concentrations can be high and superspreading can occur. These include the cruise ship outbreak in February 2020^8^ and the choir practice in Skagit county, Washington, in March 2020.^9^ Alarmingly, around 40%–45% of infected individuals are asymptomatic, where viral replication occurs quickly but unnoticed and a large number of virus-laden droplets can be exhaled. This not only increases the risk of infection to surrounding peoples but also makes it extremely difficult to contact-trace the infected and exposed individuals. In symptomatic patients, 59%–82% of people experience coughs in addition to shortness of breath, sore throat, nasal congestion, diarrhea, and vomiting, adding more viral loads to the surroundings.^10^

With every single breath, cough, or sneeze, the human lung exhales a significant amount of droplets. Respiratory droplets can be generated in different sites of the respiratory tract: the bronchioles, larynx, and oral lips, which are referred to as the B mode, L mode, and O mode, respectively.^11^ Droplets of different sizes have been measured from different origin sites, with the mean diameter being 1.8 *μ*m from the bronchioles, 3.8 *μ*m from the larynx, and 374 *μ*m from the oral lips.^11^ Using an aerodynamic particle sizer (APS), Yang *et al.* measured coughed droplets and reported a range of 0.62–15.9 *μ*m with a multimodal size distribution.^12^ Using a laser diffraction system, Zayas *et al.* reported a droplet size range of 0.1–900 *μ*m from voluntary coughs with sub micrometer droplets accounting for 97% of the total number.^13^ The large droplets of 50–100 *μ*m captured on glass slides and a microscope by Xie *et al.* might be predominately generated from the oral lips (i.e., the O mode).^14^ Similarly, Zhu *et al.* reported 30–500 *μ*m saliva droplets produced by coughs with exiting speeds up to 20 m/s.^15^ Lindsley *et al.* quantified cough droplets from influenza patients during and after illness and found significantly higher aerosol volume per cough during illness (38.3 pl) than after recovery (26.4 pl).^16^ Moreover, much of the viral RNA was contained within droplets in the respiratory size range.^17^ In small peripheral airways (i.e., B-mode), reopening of the collapsed terminal airway structures at the beginning of the inhalation is the major mechanism of droplet generation.^18–20^ These droplets were transported into the pulmonary alveoli during inhalation and were exhaled with expiratory airflows in terms of either breathing, coughing, sneezing, talking, or singing. The alveolar region is one of the three susceptible binding sites for SARS-CoV-2 viruses; it is also the final disease site where the type II cells are attacked, and viruses quickly replicate.^21,22^ Pneumonia is typical in severe COVID-19 patients, where body fluids accumulate in the alveoli, intensifying aerosol generation. Moreover, inflammation in the respiratory bronchioles will increase the probability of airway collapse and opening, further increasing virus-laden aerosol generation. As a result, droplets exhaled from the alveoli can be more infectious than droplets from other airway sites. The knowledge of the exhaled fraction (EF) of respiratory droplets from the alveoli, as well as their sizes and exiting speeds, can be highly useful in the management of COVID-19 transmission.^23,24^

However, such information is severely lacking due to many challenges, such as the inaccessibility to deep lungs for *in vivo* measurement and visualization, the extremely low amounts of collected analytes in picogram per liter of exhaled air,^18^ and the complex respiratory geometry from the alveoli to the mouth that makes it impractical to conduct *in vitro* or numerical studies in the entire respiratory tract geometry. If modeled in 3D, prohibitively large file and mesh size are required to resolve the entire respiratory tract, which is still infeasible in today's computing capacity available in labs. For instance, there are 300 × 10^6^–600 × 10^6^ alveoli in adult lungs that compose a total surface area of more than 72 m^2^.^25^ Mallik *et al.* experimentally characterized the inspiratory deposition of nebulized aerosols in tubes of 0.3–2 mm diameters that represented lung bronchioles of different generations and reported that long inhalation and subsequent breathing hold both enhanced particle deposition and the chance of viral infection.^26^ No analytical or numerical studies that investigated the process of the respiratory droplets being exhaled from the alveolar region (deep lung) to the mouth have been reported. Therefore, it is not easy to differentiate the source sites of exhaled aerosols, the relative fractions from each site, and the size distribution and exiting speeds of the droplets from different source sites.

The objective of this study is to numerically model and simulate the process of the alveolar droplets being exhaled from the alveolar region through all bifurcation generations to be exhaled through the mouth opening. A whole airway model was developed that comprised an upper airway, an alveolar model, and a single-path lung from G3 to G18 following Weibel's demographic dimension.^27^ An experimentally measured cough waveform was used to control the alveolar wall motion and the tidal flows at the lung branches of each generation.^28^ The mouth opening will be modeled after a cough image captured using a high-speed camera.^29^ Specific aims include:

(1) Simulate the expiratory airflow and particle motions from the alveolar through the respiratory tract.
(2) Quantify the fraction of depletes being exhaled from the mouth, as well as the exiting velocity distributions, for particles ranging from 0.1 to 4 *μ*m.
(3) Quantify the deposition fraction (DF) in different regions of the respiratory tract, including the alveoli, lung branches, and upper airway.
(4) Compare the droplet exhalation fraction and existing velocities at different cough depths.

## II. METHODS

### A. Study design

**FIG. 1.**
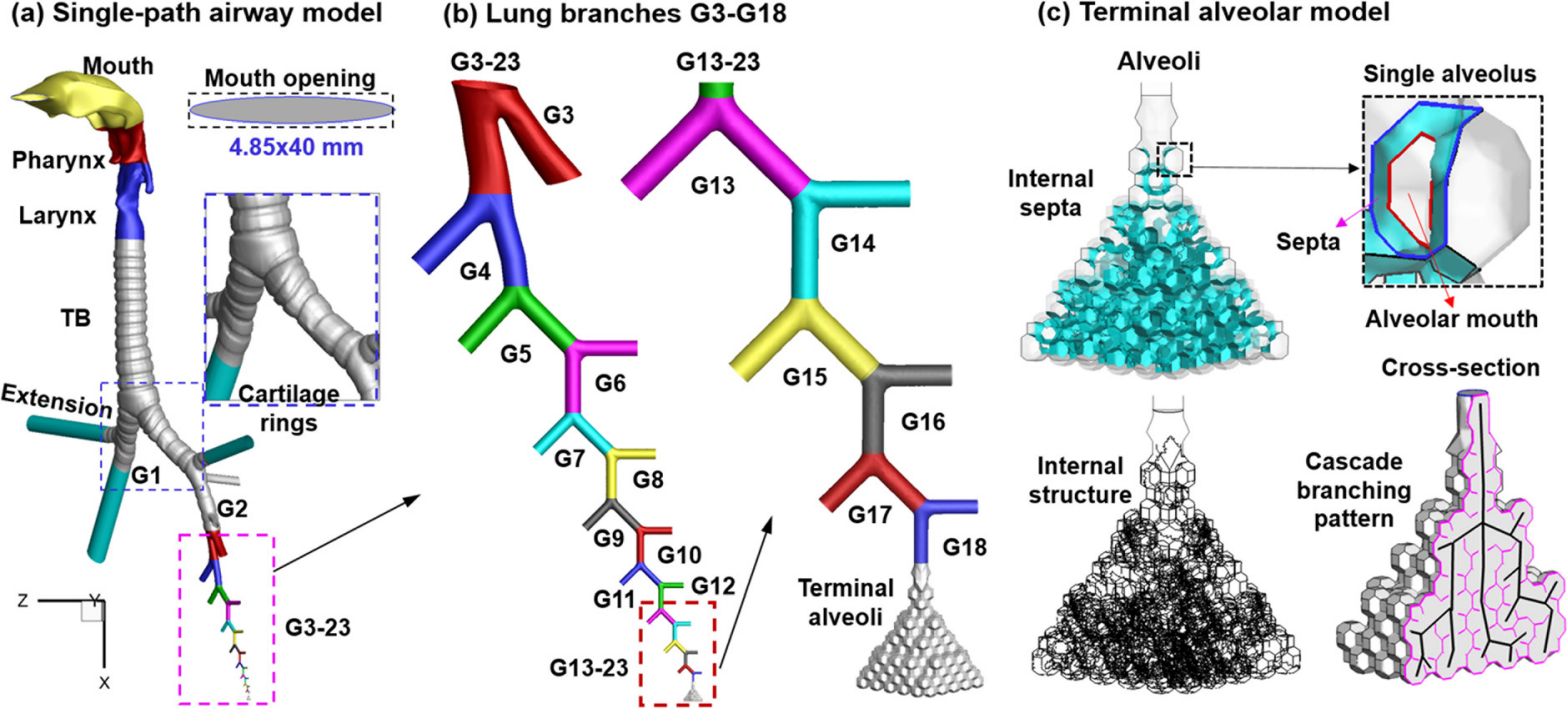
Multiscale single-path whole lung model: (a) single-path airway model from the mouth to the terminal alveoli, (b) single-path lung branches from G3 to G23, and (c) terminal alveolar model with internal septa and a cascade-branching pattern of alveolar ducts. The mouth opening has an ellipse ship and a dimension of 4.85 × 40 mm^2^, as captured using a high-speed camera during a cough. Cartilage rings are retained in the trachea, as well as in the main and secondary bronchi (i.e., G1 and G2).

**FIG. 2.**
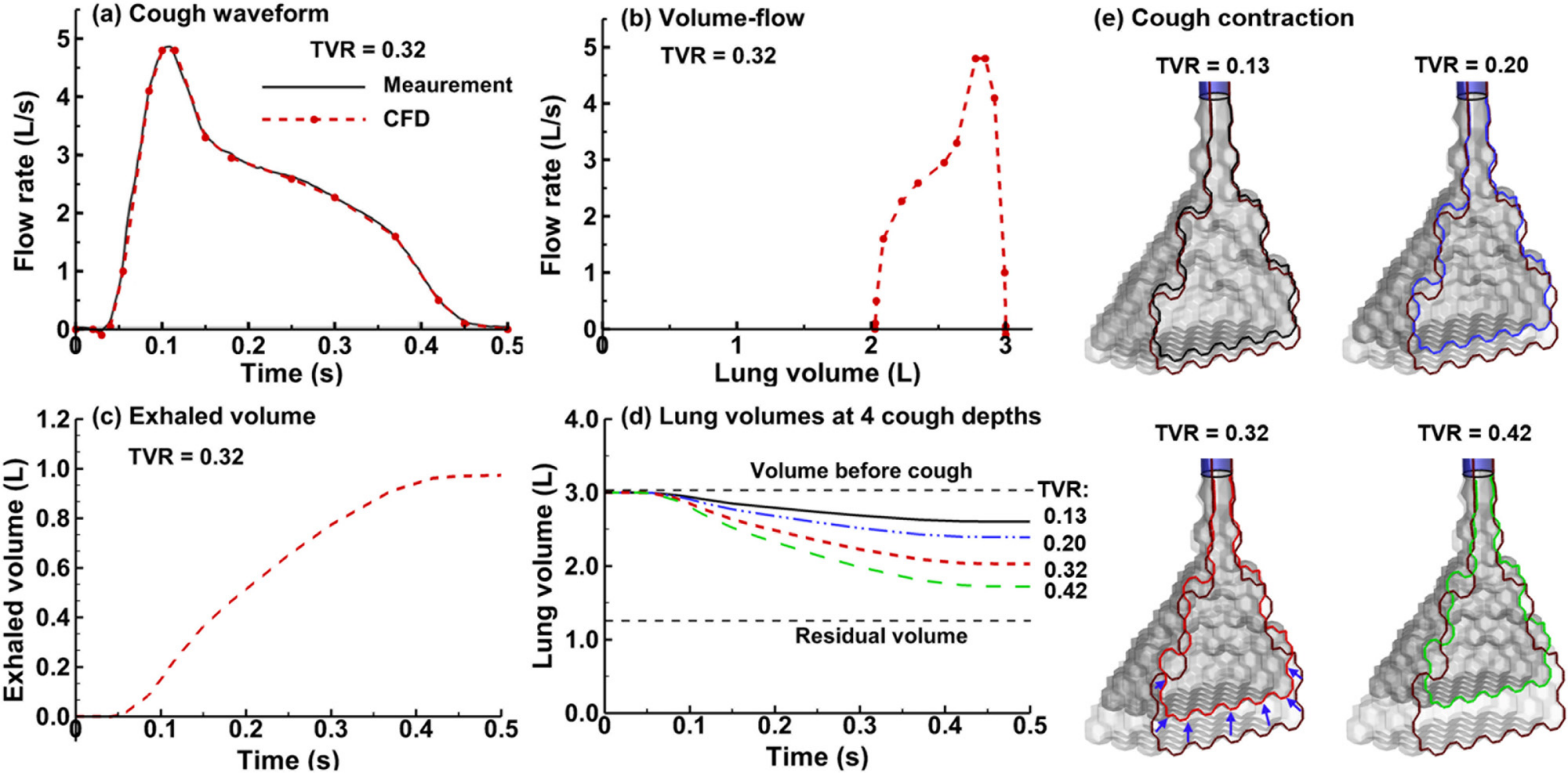
Cough waveform and alveolar wall kinematics: (a) the *in vivo* measured cough waveform^28^ vs the computationally implemented (CFD: computational fluid dynamics) waveform represented using 15 line segments, (b) the volume-flow profile of the cough, (c) the time-varying exhaled volume, and (d) the time-varying lung volumes at four cough depths. The alveolar volumes at the start and end of a cough are shown in (e) at four cough depths. TVR: tidal volume ratio.

A single path whole lung model that extended from the mouth opening to the terminal alveoli was developed, as shown in Fig. 1(a). It was a combination of an existing mouth-lung model extending to G3,^30^ a newly developed single-path lung branch geometry from G4 to G18 [Fig. 1(b)] based on Weibel's symmetric lung dimensions (Table I),^27^ and an alveolar model comprising four generations (G19–G23) of alveolar ducts [Fig. 1(c)]. To evaluate the exiting velocities of exhaled droplets, the morphology and dimension of the mouth opening were modified according to cough images captured using a high-speed camera.^29^ Droplets were spatially distributed in the alveolar region in a stochastic manner before coughing to represent droplets generated during inhalation via the bronchiolar-collapse-opening mechanism.^18^ These droplets were expelled to the mouth by the alveolar wall contraction following an experimentally measured cough waveform [Figs. 2(a)–2(d)].^28^ Transient velocity profiles synchronized with the alveolar wall motion and consistent with the generation-specific ventilation partition were specified at the single-path branch openings of G3–G18 [Fig. 1(b)]. Droplets being retained in the alveoli, lung bifurcations, and extrathoracic airway, as well as droplets being exhaled, were quantified. To study the effects of the size of respiratory droplets that were generated in peripheral airways, monodisperse aerosols of 0.1, 0.4, 0.8. 1, 2, 3, and 4 *μ*m diameter were considered separately, with the size range following the measurements of Johnson *et al.*^11^ and Bake *et al.*^18^ To study the influences from the cough depth (breath-holding) on exhaled droplet fractions, four tidal volume ratios (TVR = 0.13, 0.2, 0.32, and 0.42) were simulated [Figs. 2(d) and 2(e)]. The TVR is defined as the ratio of the cough-exhaled volume over the lung volume before the cough. More details of the model development, dynamic boundary conditions, and numerical methods are presented later.

**TABLE I.** The morphological dimension and meshing parameters of the single-path lung. There are four layers of prismatic mesh in the near-wall regions with a height ratio of 1.3.

Generation (*Z*)	Number per generation, *n*(*Z*)	Diameter *d*(*Z*) (mm)	Length *l*(*Z*) (mm)	Maximal mesh size (mm)	Near-wall mesh size (mm)
4	16	4.5	12.7	0.20	0.018
5	32	3.5	10.7	0.16	0.017
6	64	2.8	9.0	0.15	0.016
7	128	2.3	7.6	0.14	0.015
8	256	1.86	6.4	0.13	0.014
9	512	1.54	5.4	0.12	0.013
10	1024	1.3	4.6	0.11	0.012
11	2048	1.09	3.9	0.10	0.011
12	4096	0.95	3.3	0.09	0.010
13	8192	0.82	2.7	0.08	0.009
14	16 384	0.74	2.3	0.07	0.008
15	32 768	0.66	2.0	0.06	0.007
16	65 536	0.6	1.65	0.05	0.006
17	131 072	0.54	1.41	0.04	0.005
18	262 144	0.5	1.17	0.03	0.004

### B. Single-path whole lung model and kinematics

#### 1. Mouth-lung model till G18

The mouth-lung airway model extending to G3 was previously developed by Xi and Zhao^30^ and is a combination of three previous models: a cast-based oral cavity with a modified mouth opening, a CT (computed tomography) based pharyngolaryngeal airway, and a cast-based tracheobronchial geometry. The oral cavity model in the mouth-lung geometry was previously reconstructed from oral airway cast reported by Cheng *et al.*,^31^ which, along with its variants, has been used in many numerical and experimental dosimetry studies.^32–34^ To simulate the exiting speeds of exhaled droplets at the mouth opening, an image of the lip position captured using a high-speed camera was adopted, which has an ellipse shape and a dimension of 4.85 × 40 mm^2^ [upper inset of Fig. 1(a)].^29^ The pharyngolaryngeal model was segmented from CT head scans of a 53-year-old adult.^35,36^ The tracheobronchial geometry was developed from an anatomical replica.^37^ C-shaped cartilage rings were retained in the trachea till G3 [inset of Fig. 1(a)], which prevent airway collapse.^38^ The length of the trachea is 90 mm and the diameter is 19 mm. The lengths of the left and right main bronchi were 57.5 and 23 mm, respectively, and the diameters of the two bronchi were 14.1 and 14.3 mm, respectively [Fig. 1(a)].

From G4 to G18, a single path lung bifurcation geometry was generated following the Webels's lung morphology [Fig. 1(b)], with the diameter and length of each generation listed in Table I.^27^ The bifurcation angle in G4–G18 was 90°. As the first approximation, the single-path lung structure (G4–G18) stemmed from the left lower segmental bronchus and extended into the base of the left lower lobe, with all bifurcations in one corona plane. Single-path lung structures with different dimensions and arrangements can be considered following the same method in future studies. The last bronchiole (G18) is connected to an alveolar model, where the droplets were stored before cough and whose quick wall contraction dispensed the aerosols toward the mouth opening during the cough.

#### 2. Alveolar model with inter-alveolar septa and wall motion

An alveolar model comprising 496 alveoli and 62 alveolar ducts [Fig. 1(c)] was generated using an in-house module Lung4Cer.^39,40^ Each alveolus was represented using a polygon. The inset of Fig. 1(c) illustrates an individual alveolus that is connected to the main alveolar duct via an octagonal mouth (red). There are inner septal walls shared by the alveolus and the alveolar ducts (cyan color). The alveolar model has a branching structure where the daughter alveolar ducts bifurcate from the main (parent) alveolar duct and form a complex network of inter-alveolar septal walls organized in a pyramid-shaped space [lower inset of Fig. 1(c)]. In other words, the 496 alveolar units are not simply clustered around the main duct; instead, they belong to 62 different daughter alveolar ducts, whose position and orientation are determined based on the space-filling algorithm developed by Kitaoka *et al.*^41,42^

#### 3. Alveolar wall kinematics and branch boundary conditions

Kinematics of the alveolar wall motion was specified following the experimentally measured waveform of the cough flow rate,^28^ which was characterized by a cough peak flow rate (CPFR) of 4.8 l/s, a cough expired volume (CEV) of 0.97 l, and a peak velocity time of 0.103 s, as shown by the black line in Fig. 2(a). In comparison to a normal breathing profile with 2.5–3.0 s exhalation, a typical cough has an expiratory phase of 0.5 s. The peak velocity is also much higher during cough, i.e., at 4.8 l/s (or 288 l/min) in comparison to 0.25 l/s (or 15 l/min) during normal exhalation. The cough profile used in simulations was represented using 16 points [red dashed lines in Fig. 2(a)], whose values were listed in Table II. Based on the cough waveform, a volume of 0.97 l is exhaled, giving a tidal volume ratio (TVR) of 0.32 based on an initial lung volume of 3 l at the beginning of the cough.

**TABLE II.** Approximated and normalized CFD cough profiles. *f*(*t*): Normalized cough profile.

Acceleration phase				Deceleration phase			
Point (*Z*)	Time (*s*)	Vel (*L*/*s*)	Normalized	Point (*Z*)	Time (*s*)	Vel (*L*/*s*)	Normalized
1	0	0	0.000	9	0.15	3.3	0.688
2	0.02	0	0.000	10	0.18	2.95	0.615
3	0.03	−0.1	−0.021	11	0.25	2.59	0.540
4	0.04	0.05	0.010	12	0.3	2.27	0.473
5	0.055	1	0.208	13	0.37	1.6	0.333
6	0.085	4.1	0.854	14	0.42	0.5	0.104
7	0.1	4.8	1.000	15	0.45	0.1	0.021
8	0.115	4.8	1.000	16	0.5	0	0.000

The transient boundary condition at the bronchiolar and bronchial opening follows the cough flow waveform,^28^ with the magnitude being generation specific and consistent with the flow partition through that branch. For instance, at G10, there are 1024 (210) bronchioles in the lung, and each G10 bronchiole has a volumetric flow rate of one *Q*(*t*)/1024 (l/s), where *Q*(*t*) = 4.8 × *f*(*t*) (l/s), with 4.8 l/s being the peak flow rate and *f*(*t*) being the normalized cough waveform as listed in Table II.^28^ Based on a surface area of the G10 bronchiolar cross-sectional area of 1.33 mm^2^, the flow speed was specified as 3.52 × *f*(*t*) (m/s). Similarly, synchronized, transient velocity boundary conditions were specified at each branch opening from G18 to G1 (main bronchi) based on the generation-specific flow partition and branch cross-sectional area. As a result, the total volumetric flow rate summing all branches exactly matched the measured cough waveform [Fig. 2(a)]. User-defined C-modules were developed to prescribe the alveolar wall contraction and synchronized time-varying velocity boundary conditions at the branch opening for G3–G18.^43^

To study the influences from the depths of the cough, three different tidal volume ratios (0.13, 0.20, and 0.42) were considered in addition to the control case (0.32).^28,44^ The alveolar wall moves either slower or faster following the same normalized waveform within a fixed period of 0.5 s, so that the alveolar volume contraction was 0.13, 0.20, and 0.42 times the initial volume [Fig. 2(e)]. The velocity boundary condition at the branch ends from G18 to G0 was scaled by a constant factor [e.g., a factor of 0.406 25 (=0.13/0.32)] for the tidal volume ratio of 0.13.

### C. Computational fluid-particle dynamics (CFPD)

Isothermal (37 °C) and incompressible (ρ = 1.139 kg/m^3^) air were assumed for the expiratory airflows during the cough. Considering the multiscale dimensions of the respiratory system that spanned three orders of magnitude (i.e., from the upper airway with a hydraulic diameter of 20 mm to the alveolar diameter of 0.2 mm), multiple flow regimes were expected. The low Reynolds number (LRN) *k-ω* turbulence model was adopted for its demonstrated capacity in capturing the turbulent–laminar transitions in both the main flow and the near-wall regions.^45^ The governing equations for the mass and momentum conservations are^46^

$$\frac{\partial u_{i}}{\partial x_{i}}=0;\frac{\partial u_{i}}{\partial t}+u_{j}\frac{\partial u_{i}}{\partial x_{j}}=-\frac{1}{\rho }\frac{\partial p}{\partial x_{i}}+\frac{\partial }{\partial x_{j}}\left[\left(\nu +\nu_{T}\right)\left(\frac{\partial u_{i}}{\partial x_{j}}+\frac{\partial u_{j}}{\partial x_{i}}\right)\right],$$ (1)

where $u_{i}$ and $u_{j}$ are the fluid velocity components, *ρ* is the fluid density, *ν* is the kinematic viscosity, *p* is the pressure, and *ν_T_* is turbulent viscosity defined as $\nu_{T}=\alpha^{*}k/\omega$. The parameter α* is evaluated as^46^

$$\alpha^{*}=\frac{0.024+k/6\nu \omega }{1.0+k/6\nu \omega }.$$ (2)

For laminar flow, $\nu_{T}$ is zero. The turbulent kinetic energy (*k*) and the specific dissipation rate (*ω*) are governed by^46,47^

$$\frac{\partial k}{\partial t}+u_{j}\frac{\partial k}{\partial x_{j}}=\tau_{ij}\frac{\partial u_{i}}{\partial x_{j}}-\varepsilon_{k}+\frac{\partial }{\partial x_{j}}\left[\left(\nu +0.5\nu_{T}\right)\left(\frac{\partial k}{\partial x_{j}}\right)\right],$$ (3)

$$\frac{\partial \omega }{\partial t}+u_{j}\frac{\partial \omega }{\partial x_{j}}=\frac{13}{25}\frac{\omega }{k}\tau_{ij}\frac{\partial u_{i}}{\partial x_{j}}-\varepsilon_{\omega }+\frac{\partial }{\partial x_{j}}\left[\left(\nu +0.5\nu_{T}\right)\left(\frac{\partial \omega }{\partial x_{j}}\right)\right],$$ (4)

where *τ_ij_* is the shear stress tensor, ε_k_ is the dissipation of *k*, and ε_ω_ is the dissipation of *ω*.^46^ Li *et al.* evaluated the performances of different turbulence models in simulating respiratory flows against *in vitro* measurements and observed that the LRN *k-ω* model provided the best approximation among four Reynolds-averaged Navier–Stokes (RANS) turbulence models (i.e., *k-ε*, LRN *k-ω*, shear stress transport (SST) *k-ω*, and Reynolds Stress Model).^48^

Particles were tracked during the cough (expiratory phase) for one cycle. A well-tested Lagrangian-tracking model was used to simulate the particle motion and deposition in the respiratory tract, whose transport equations are^47^

$$\frac{dv_{i}}{dt}=\frac{f}{\tau_{p}}\;\left(u_{i}-v_{i}\right)+g_{i}(1-\alpha )+f_{i,\;\mathrm{lift}}+f_{i,\mathrm{Brownian}};\begin{array}{cc} & \frac{dx_{i}}{dt}=v_{i}(t) \end{array},$$ (5)

where *u_i_* and *v_i_* are the fluid and particle velocity components, respectively, *g_i_* is the gravity, *τ_p_* is the characteristic time required for a particle to respond to changes in fluid motion expressed as *τ_p_* = C_c_*ρ_p_d_p_*^2^/18*μ*, *C_c_* is the Cunningham correction factor for diffusive droplets based on the expression of Allen and Raabe,^49^ and *f* is the drag coefficient based on the expression of Morsi and Alexander.^50^ The four terms at the right-hand side of the first equation in Eq. (5) represent the aerodynamic drag force, gravitational sedimentation, Saffman lift force, and Brown motion force, respectively. The Brownian motion force is expressed as:^47^

$$f_{i,\mathrm{Brownian}}=\frac{\varsigma_{i}}{m_{d}}\sqrt{\frac{1}{\tilde{D}}\frac{2{k_{B}}^{2}T^{2}}{\Delta t}}\begin{array}{cc} ; & \tilde{D}=\frac{k_{B}TC_{c}}{3\pi \mu d_{p}} \end{array},$$ (6)

where *ς_i_* is a zero-mean Gaussian probability density function, *m_d_* is the droplet mass, $\tilde{D}$ is the Brownian diffusion coefficient, *k_B_* = $1.38\times 10^{-16}$ cm^2^ g/s is the Boltzmann constant, Δ*t* is the time step, T is the body temperature (310.15 K), and *d_p_* is the droplet diameter. Droplets deposit on the respiratory tract when they contact the wall. This discrete-phase Lagrangian model was also enhanced with near-wall velocity interpolations for both airflow (velocity and turbulence anisotropy) and particles,^35,51^ which had been proved to provide accurate predictions of the measured dosimetry for aerosols at both nano-^52,53^ and microscales.^54,55^

### D. Numerical methods

**FIG. 3.**
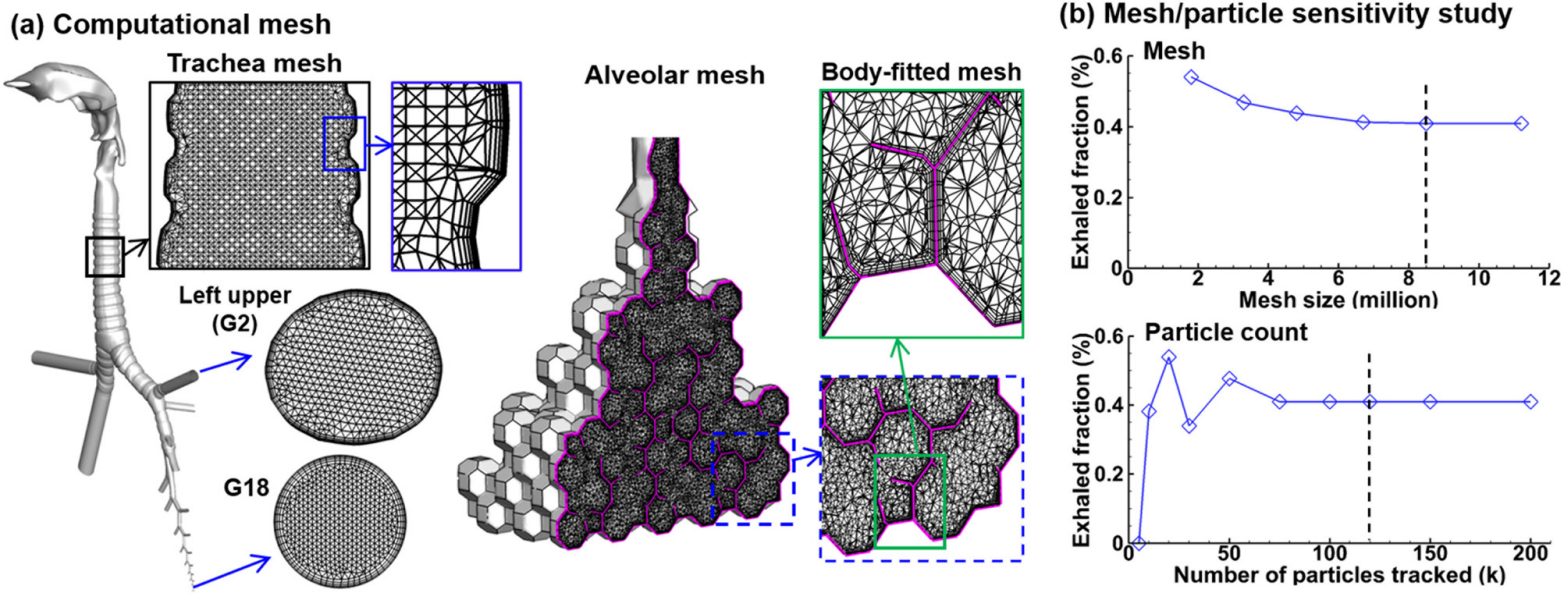
Computational mesh and sensitivity studies: (a) multiscale computational mesh with fine body-fitted elements in the near-wall region throughout the airway and (b) mesh sensitivity study of the exhalation fraction of 1-*μ*m droplets by varying the mesh size from 1.8 × 10^6^ to 11.2 × 10^6^, and particle count sensitivity study by varying the number of tracked particles from 5000 to 200 000 for a given mesh of 8.5 × 10^6^.

ANSYS Fluent 19.1 (Canonsburg, PA) was used to resolve tidal respiratory flows and track particle trajectories. In-house programs were written to control the alveolar wall motions and record the temporal-spatial deposition of droplets.^32^ One-way structure-fluid-particle interaction was assumed, i.e., from the moving alveolar wall to airflow to entrained droplets. ANSYS ICEM CFD (Canonsburg, PA) was utilized to generate the computational mesh. To adequately resolve the multiscale dimension of the whole lung model, multidomain meshes were created with different element sizes in different regions of the lung [Fig. 3(a)]. Body-fitted prismatic meshes were specified in the near-wall region throughout the single-path whole lung model. The meshing parameters in the multiscale single-path lung model are listed in Table I, with the near-wall height of G18 being 0.004 mm (4 *μ*m). In particular, the prismatic mesh was applied to both one-side outer walls and two-side septal walls, with four layers of body-fitted cells and a height of 2.5 *μ*m in the first layer cell [Fig. 3(b)]. The insets of Fig. 3(a) show the prismatic mesh zoomed at different scales, which clearly shows the high-resolution mesh near the wall and the relatively coarse mesh in the flow domain. Our previous studies have demonstrated that a prismatic mesh in the near-wall region is essential to obtain accurate inhalation dosimetry in comparison to experimental inhalation dosimetry for both submicrometer^47,56,57^ and micrometer^53,58^ aerosols.

To secure accurate inhalation dosimetry predictions, sensitivity analyses were performed in two steps [Fig. 3(b)]. First, the grid-independent study was conducted by comparing six meshes from very coarse (1.83 × 10^6^ cells) to very fine (11.94 × 10^6^ cells), all with a four-layer prismatic mesh at the first layer. The exhaled fraction (EF) of 1-*μ*m droplets from the mouth was compared, as shown in the upper panel of Fig. 3(b). It is observed that a coarse mesh gave rise to a higher value. The EF became stable at the mesh size of 8.5 × 10^6^, with negligible variation (<1%) when further refining the mesh to 11.94 × 10^6^ cells. Thus, the computational mesh with 8.5 × 10^6^ cells was adopted for all subsequent test cases. In the second step, the number of particles necessary to obtain stable dosimetry results was determined by testing 10 groups of aerosols that consisted of 5k, 10k, 20k, 30k, 50k, 75k, 100k, 120k, 150k, and 200k of 1-*μ*m particles, respectively [lower panel of Fig. 3(b)]. With an insufficient amount of seed particles (e.g., 5k), no droplets were predicted to exit the mouth opening. With incrementally more seed particles, fluctuation in the exhalation fraction was observed in the range of 10k–50k, presumably due to the complex interactions between particles and multiscale respiratory passages. This nonmonotonic variation emphasizes the need for a sufficient number of sample particles to achieve statistically invariant results. The exhalation fraction reached a stable value at 120k and beyond. So that 120k seed particles were used in this study so that the variance in exhalation fraction can be minimal as possible.

The contracting wall motion of the alveolar wall was controlled using the dynamic mesh module with a time step of 0.005 s. Synchronized, transient flow boundary condition was prescribed at each branch from G2 to G18 with a generation-specific flow rate that altogether matches the experimentally measured cough flow rate at the mouth opening. With a mesh size of 8.5 × 10^6^ and a seed particle count of 120k, one test case (i.e., one cough cycle and one particle size) took around 260 h in an AMD Ryzen 9 3960× 24-Core workstation with 256 G RAM and 3.79 GHz frequency.

## III. RESULTS

### A. Cough airflow dynamics

**FIG. 4.**
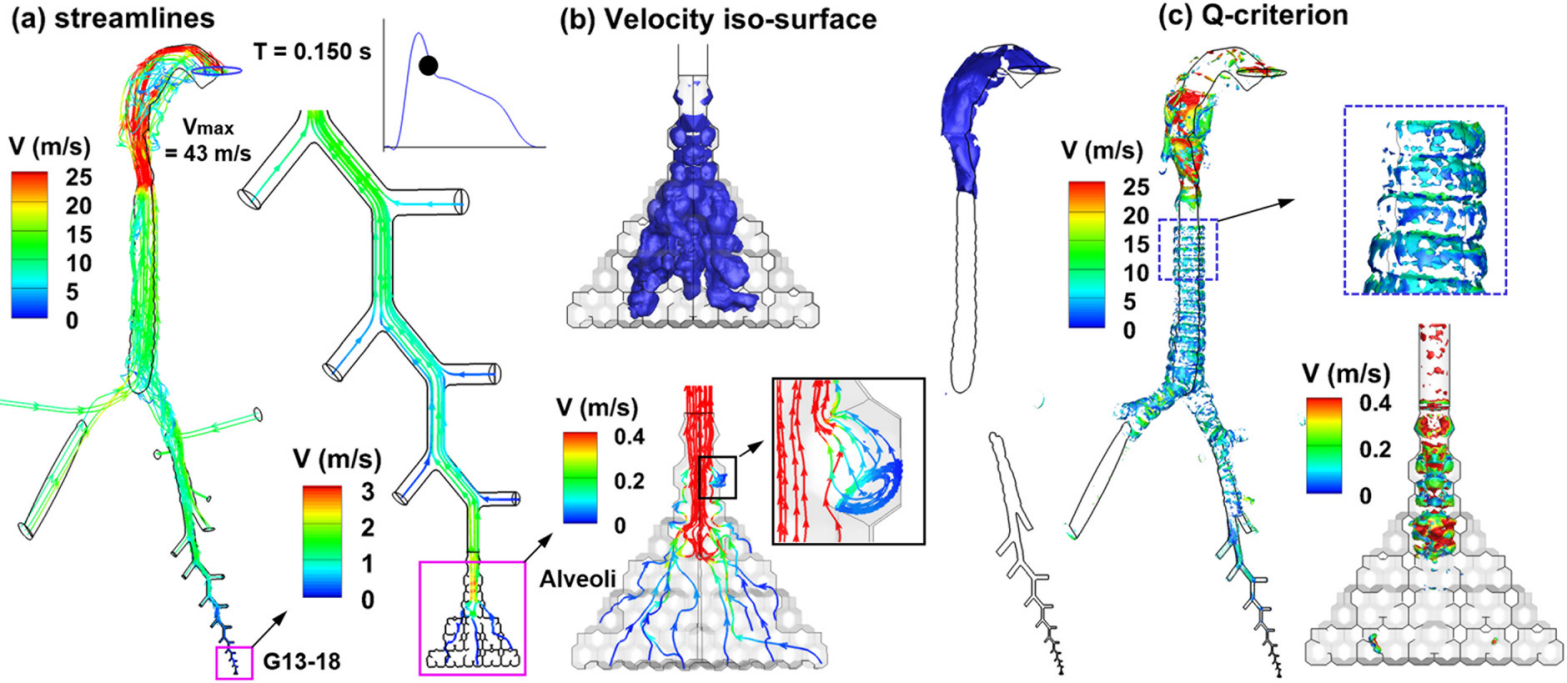
Cough airflows at T = 0.15 s in the respiratory tract: (a) streamlines, (b) velocity iso-surface, and (c) instantaneous coherent structures in terms of the Q-criterion.

The cough airflows are illustrated in Fig. 4 in terms of streamlines, velocity iso-surfaces, and vortex structures at 0.15 s from the beginning of the cough. The volumetric flow rate at this instant is 3.3 l/s [Fig. 2(a)]. Note that different ranges were used for flow visualization in different regions of the airway, which varied by two orders of magnitudes. Figure 4(a) shows the streamlines from the pulmonary alveoli to the mouth. Flows from different generations of bronchioles merged into the main flow, leading to a progressive increase in flow velocity in larger airways. The flows in the bronchioles G13–G18 are predominantly laminar, which is in direct contrast to the turbulent flow patterns in the upper airway from G2 to the mouth. In particular, highly complex stream traces are observed inside the oral cavity, which can be attributed to the laryngeal jet (red color), the flow area expansion from the pharynx to the mouth, and the flow contraction due to the narrow mouth opening. Even though a range of 0–25 m/s has been used to visualize the flows, the highest flow speed is found to be 43 m/s, which can locally lead to high-speed aerosols and highly heterogeneous transportation and deposition.

Figure 4(b) shows the velocity iso-surfaces in the alveoli (0.05 m/s) and upper airway (20 m/s). Due to the intricate network of the internal septa, the alveolar flow patterns also appear very irregular and can vary dramatically during the cough cycle. Such flow irregularities can substantially affect the alveolar retention of respiratory droplets and subsequently the exhaled fraction from the mouth opening. The vortex structures (Q-criterion) at t = 0.15 s are shown in Fig. 4(c). Anatomical details, such as cartilage rings in the trachea and inner septal walls in the pulmonary alveoli, can be predominant in inducing instantaneous coherent structures, which play important roles in aerosol mixing and transport.

### B. Particle dynamics

**FIG. 5.**
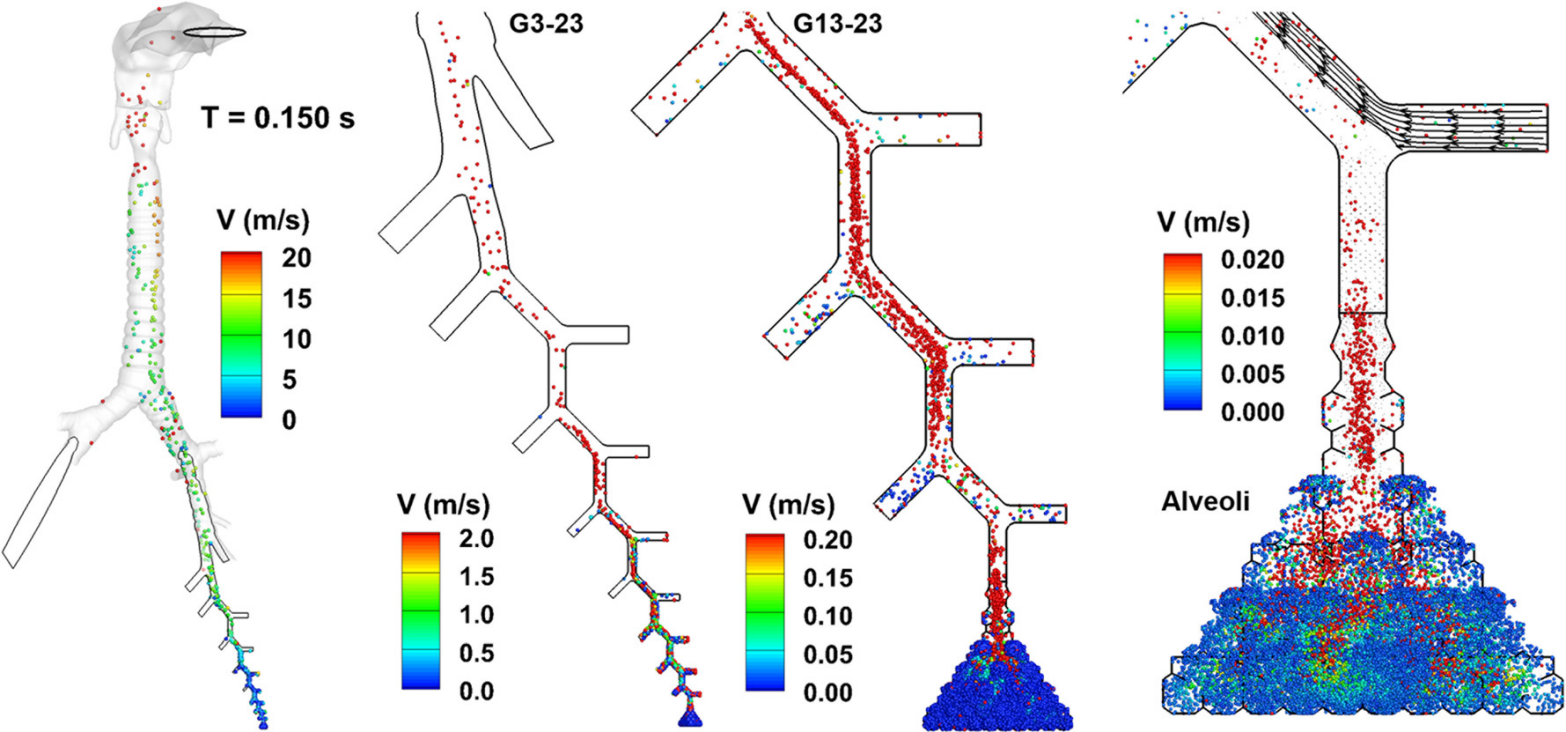
Snapshots of 1-*μ*m droplet positions at t = 0.15 s from the beginning of the cough at TVR = 0.32.

Particle dynamics during a normal cough (TVR = 0.32) was visualized using the snapshots of droplet positions at varying instants. Figure 5 displays the particle positions in different regions of the airway at 0.15 s from the beginning of the cough. It is noted that a slightly lower range of the color bar has been used to best visualize particle kinematics than those used to visualize the flows [Figs. 5 vs 4] at all sites considered (i.e., upper airway, G3–G23, and alveoli), reflecting the velocity differences between the respiratory airflow and the entrained droplets. Respiratory droplets from the pulmonary alveoli reach the mouth opening at 0.15 s. Due to the glottis-associated acceleration, droplet velocities are above 20 m/s in the mouth cavity. By contrast, the droplet velocities in the central airway (G3–G8) are on the order of magnitude of 2 m/s, those in the respiratory zone (G13–G18) 0.2 m/s, and in the pulmonary alveoli 0.02 m/s (Fig. 5). Most droplets in G13–G18 are concentrated in the core flows, a hallmark of laminar tubular flows. By contrast, droplets in the region from G6 and above fill the airway lumens. It indicates significant flow-particle mixing and enhanced circumferential dispersion in addition to the axial advection.

**FIG. 6.**
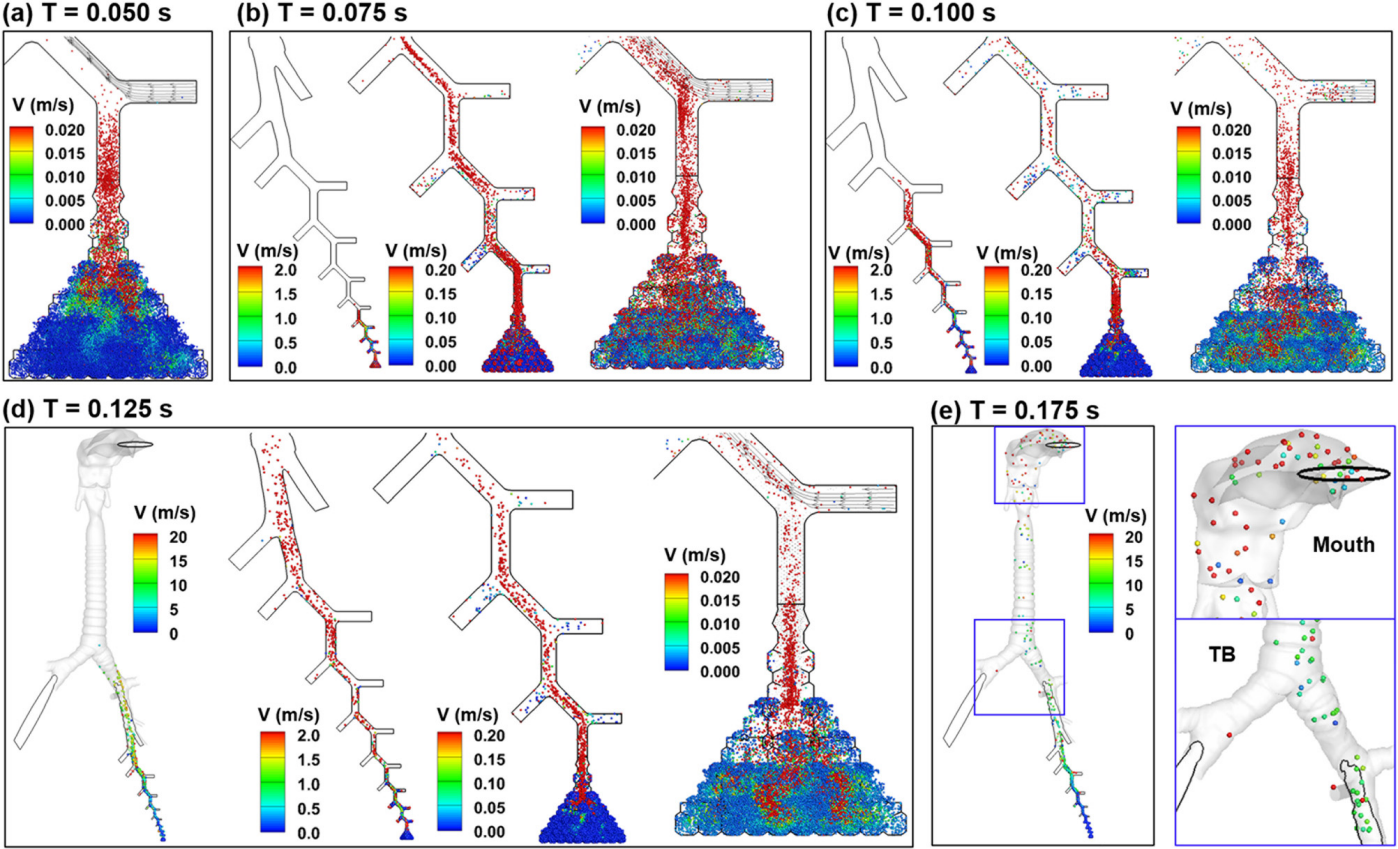
Snapshots of 1-*μ*m droplet positions at TVR = 0.32 at varying instants from the beginning of the cough: (a) 0.05 s, (b) 0.075 s, (c) 0.100 s, (d) 0.125 s, and (e) 0.175 s.

Instantaneous droplet dynamics during 0.05 s and 0.175 s of the cough is visualized in Fig. 6 using 1-*μ*m aerosols (also see supplementary material video S1). At 0.05 s, respiratory droplets start to exit from the contracting alveoli at a speed of 0.02 m/s or so. Heterogeneous flow distributions are spotted inside the alveoli due to the presence of inner septal walls. A closer examination also reveals several strains of high-speed aerosols amid nearly stagnant aerosols [green color vs blue in Fig. 6(a)], which results from the cascade-branching patterns of the alveolar ducts composing the acinar geometry. At 0.075 s, the droplets move from G18 to G12 [arrow in Fig. 6(b)]. As in Fig. 5, the droplets concentrate along the middle line of the single-path branches. This pattern reflects it is a laminar flow regime. The progressive confluence of expiratory flows from the component bronchioles also helps to restrict the exhaled droplets close to the ductal center. At 0.1 s, droplets reach G6 [Fig. 6(c)]. Meanwhile, the droplets become dispersed within G13–G18. Considering that the flow is still laminar, this dispersion may result from the flow transient effects in that a transition occurs around 0.1 s from a steep acceleration to deceleration, as shown in Fig. 2(a). At 0.125 s, droplets reach the main bronchus [Fig. 6(d)]. The speeds of the droplets within the alveoli continue to increase due to the time lag of the discrete aerosols behind the flow. Droplets start exiting the mouth opening around 0.15 s (Fig. 5) and continue throughout the rest of the cough [Fig. 6(e)].

### C. Exhaled droplets

#### 1. Exhaled droplet fraction

**FIG. 7.**
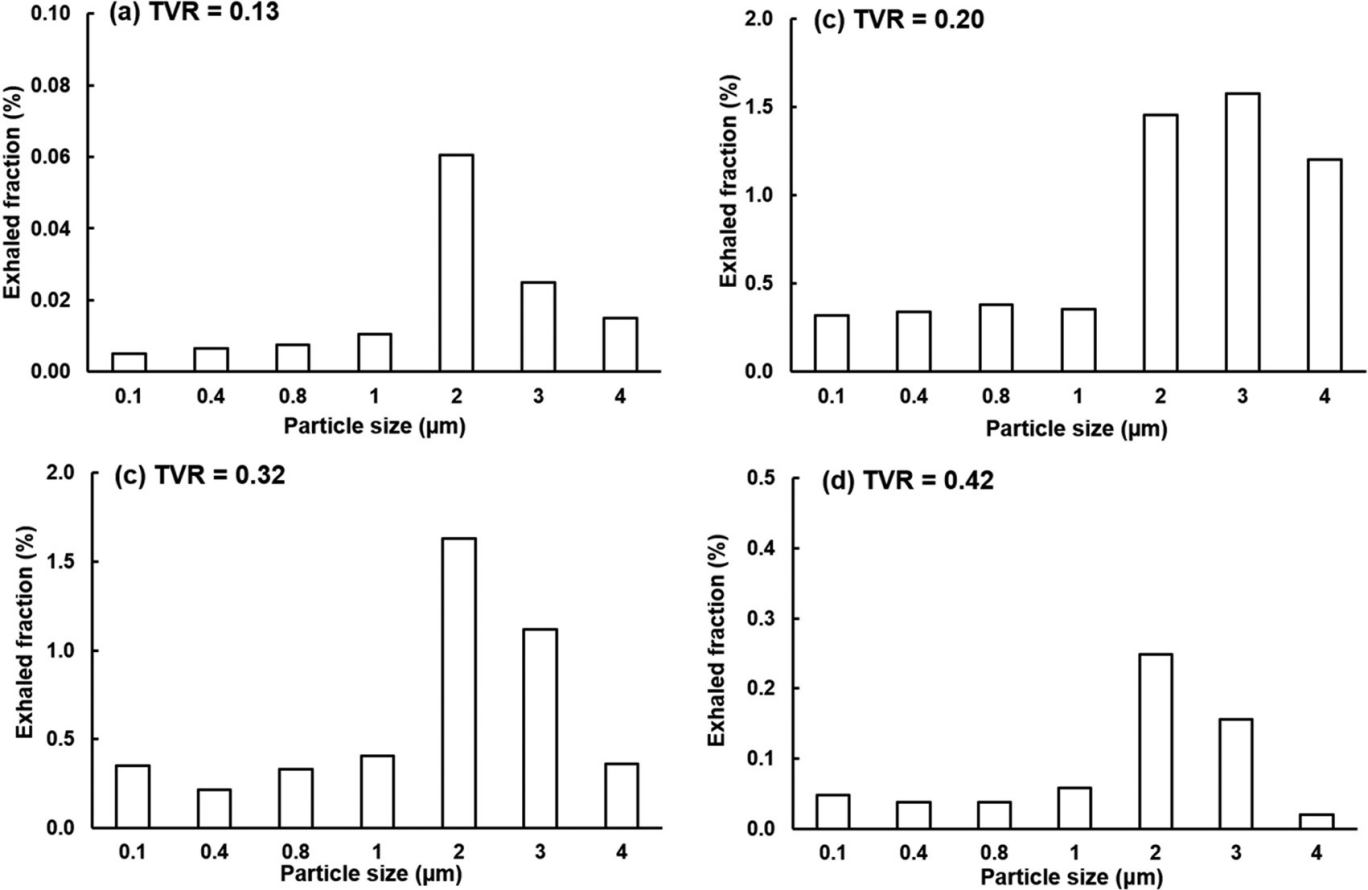
Exhaled fractions of respiratory droplets (0.1–4 *μ*m) from the pulmonary alveoli at different cough depths: (a) TVR = 0.13, (b) 0.20, (c) 0.32, and (d) 0.42.

Figure 7 compares the exhaled fractions of respiratory droplets (0.1–4 *μ*m) from the alveoli at four different cough depths, i.e., TVR = 0.13 (soft), 0.20 (moderate), 0.32 (normal), and 0.42 (vigorous). The droplet exhaled fractions are notably affected by the cough depth, which is one order of magnitude higher at TVR = 0.20 and 0.32 than those at 0.13 and 0.42. Surprisingly, increasing the cough depth does not necessarily lead to a high number of exhaled respiratory droplets. Among the four cough depths considered, the highest droplet exhaled fractions are observed at TVR = 0.32 and 0.20. For a given cough depth, the peak exhaled fraction occurs for droplets with a diameter of 2–3 *μ*m, i.e., 1.63% for 2-*μ*m droplets at TVR = 0.32 [Fig. 7(c)], 1.58% for 3-*μ*m droplets, and 1.46% for 2-*μ*m droplets at TVR = 0.32 [Fig. 7(b)].

**FIG. 8.**
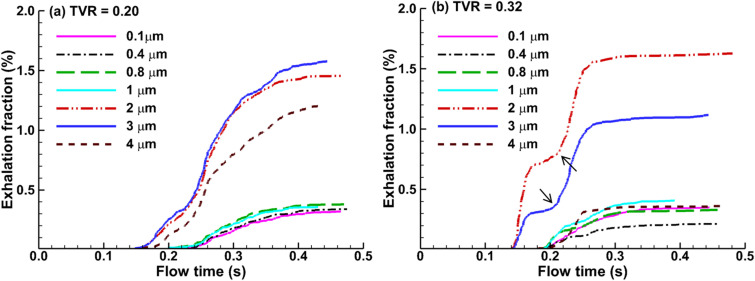
Temporal evolution of the respiratory droplet exhalation fraction for different droplet sizes at two cough depths: (a) TVR = 0.20 and (b) TVR = 0.32.

The time evolution of the droplet exhalation fraction from the mouth opening is shown in Fig. 8 for 0.1–4 *μ*m droplets. At both cough depths, micrometer droplets were exhaled earlier than the submicrometer droplets out of the mouth (about 0.05 s ahead). For all droplets considered at TVR = 0.32 [Fig. 8(b)], the exhalation fraction approached a plateau at 0.35 s, while at TVR = 0.20, the plateau was reached at the end of the cough [i.e., 0.48 s, Fig. 8(a)]. The reflection points observed for 2-*μ*m and 3-*μ*m droplets at TVR = 0.32 [black arrows, Fig. 8(b)] were presumably attributed to the reflection point in the waveform that divided the acceleration and deceleration phases [Fig. 2(a)].

#### 2. Exhaled droplet velocity distribution

**FIG. 9.**
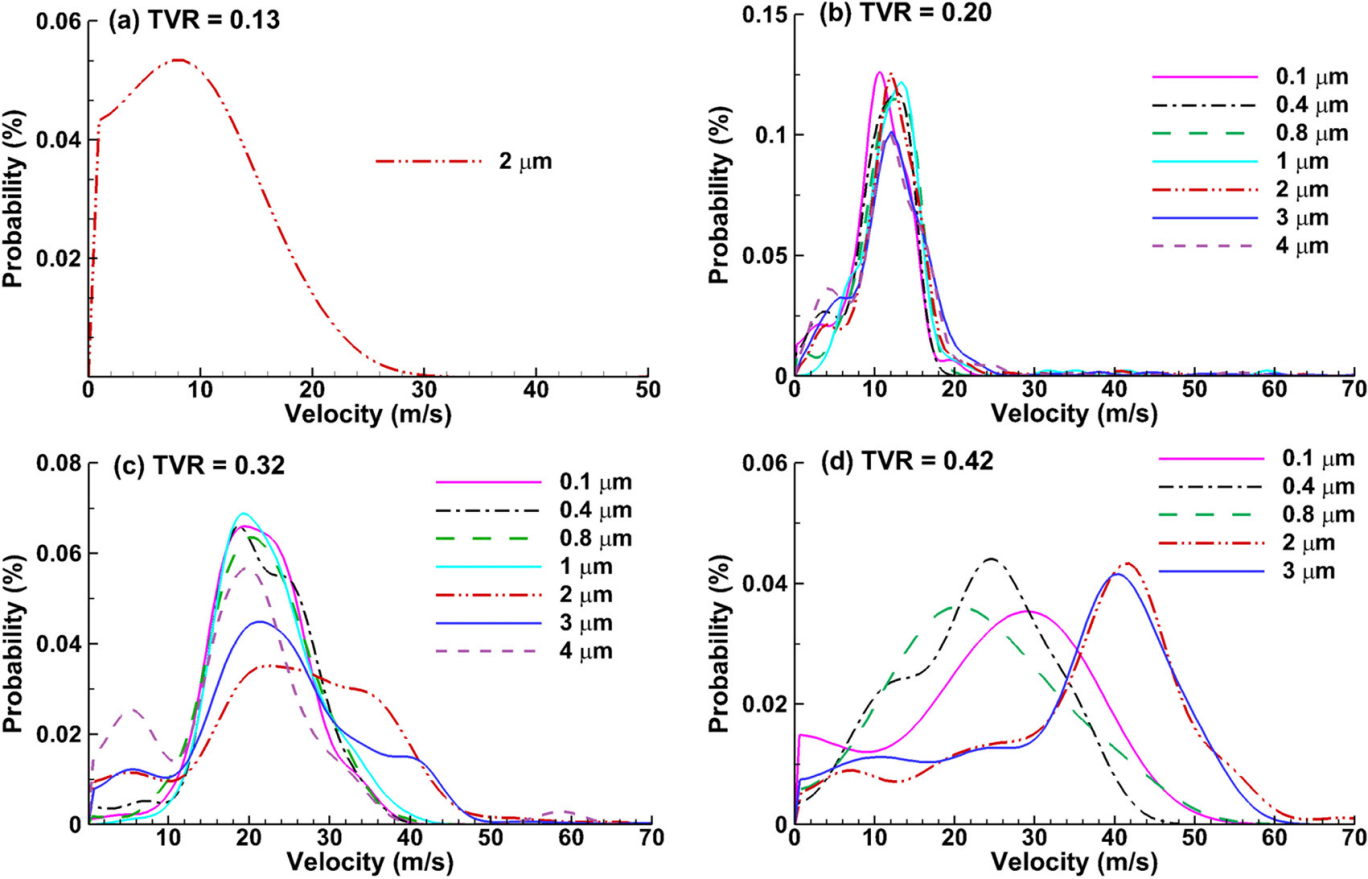
Velocity distributions of the exhaled droplets at the mouth opening at (a) TVR = 0.13, (b) 0.20, (c) 0.32, and (d) 0.42.

The exiting speeds of respiratory droplets are shown in Fig. 9 for different droplet sizes and cough depths. For the soft (TVR = 0.13) and vigorous (0.42) coughs with low exhaled droplet fractions, only particles with a statistically significant number of droplets (i.e., >70) were included. As expected, the mean velocity of exhaled droplets increases with the cough depth, which is linear with the flow rate of the carrier flow. Based on a mouth opening area of 151.6 mm^2^, the peak velocity of the main carrier flow is calculated as 12.9, 19.8, 31.7, and 41.6 m/s at TVR = 0.13, 0.20, 0.32, and 0.42, respectively. The mean velocities of exhaled droplets are consistently lower than that of the carrier flows, i.e., 9 m/s at TVR = 0.13, 12–13 m/s at TVR = 0.20, and 18–20 m/s at TVR = 0.32 [Figs. 9(a)–9(c)].

For a vigorous cough [TVR = 0.42, Fig. 9(d)], a wider range of exhaled droplet speeds are observed compared to the other three cough depths, indicating an increased nonlinearity of the particle dynamics within the respiratory tract at higher exhalation flow rates. As the exhaled speeds of the submicrometer droplets are lower than that of the carrier flow at TVR = 0.42, those of the 2–3 *μ*m droplets (40–43 m/s) are similar to those of the mean carrier flow (i.e., 41.6 m/s). It is noted that due to local accelerations and particle inertia, the droplet exhaled speeds may be higher than that of the carrier flow.

### D. Deposition in the respiratory tract

To understand the high sensitivities of the droplet exhalation fraction to the cough depth, droplets that deposit in the respiratory tract during the cough were quantified in different regions of the respiratory tract and compared between different cough depths for aerosols ranging from 0.1 *μ*m to 4 *μ*m, as detailed later.

#### 1. Terminal alveoli

**FIG. 10.**
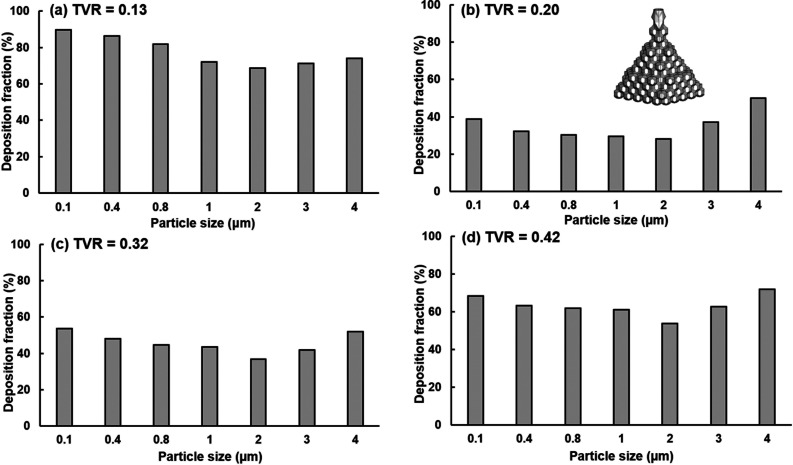
Alveolar deposition fractions at different cough depths: (a) TVR = 0.13, (b) 0.20, (c) 0.32, and (d) 0.42.

Figure 10 shows the deposition fractions (DFs) in the terminal alveoli at different cough depths. Among the respiratory droplets considered (0.1–4 *μ*m), the lowest alveolar DF occurred at 2 *μ*m for all cough depths, which is coincident with the peak exhalation fraction of 2-*μ*m droplets. Moreover, significantly different alveolar DFs are found at different cough depths [Figs. 10(a)–10(d)], which partially explains the large differences in the droplet exhalation fractions. For instance, increasing TVR = 0.13 to 0.20 remarkably decreases the alveolar deposition for all respiratory droplets considered (0.1–4 *μ*m). For 2-*μ*m droplets, the alveolar DF is 68.9% at TVR = 0.13 and 28.1% at TVR = 0.20 [Figs. 10(a) vs 10(b)]. In other words, only 21.1% of 2-*μ*m droplets escape the alveolar retention on their journey to the mouth when coughed at TVR = 0.13, while 71.9% of droplets escape at TVR = 0.20. The latter leading to more particles deposited in the respiratory tract, as well as exhaled out of the mouth.

It is also observed that increasing from TVR = 0.32 (normal cough) to 0.42 increased the alveolar DF [Figs. 10(c) and 10(d)], as opposed to the drastic decrease from TVR = 0.13 to 0.20 [Figs. 10(a) and 10(b)]. Take the example of 2-*μ*m droplets again, 36.8% and 53.7% of droplets were retained in alveoli at TVR = 0.32 (normal cough, baseline) and 0.42 (vigorous cough), respectively. However, this difference alone cannot adequately explain the one-order-of-magnitude discrepancy in the number of exhaled droplets between these two cough depths. Droplet transport and deposition along the respiratory tract should also play an important role in determining whether a droplet can be exhaled from the mouth opening.

#### 2. Single-path lung branches G3–G18

**FIG. 11.**
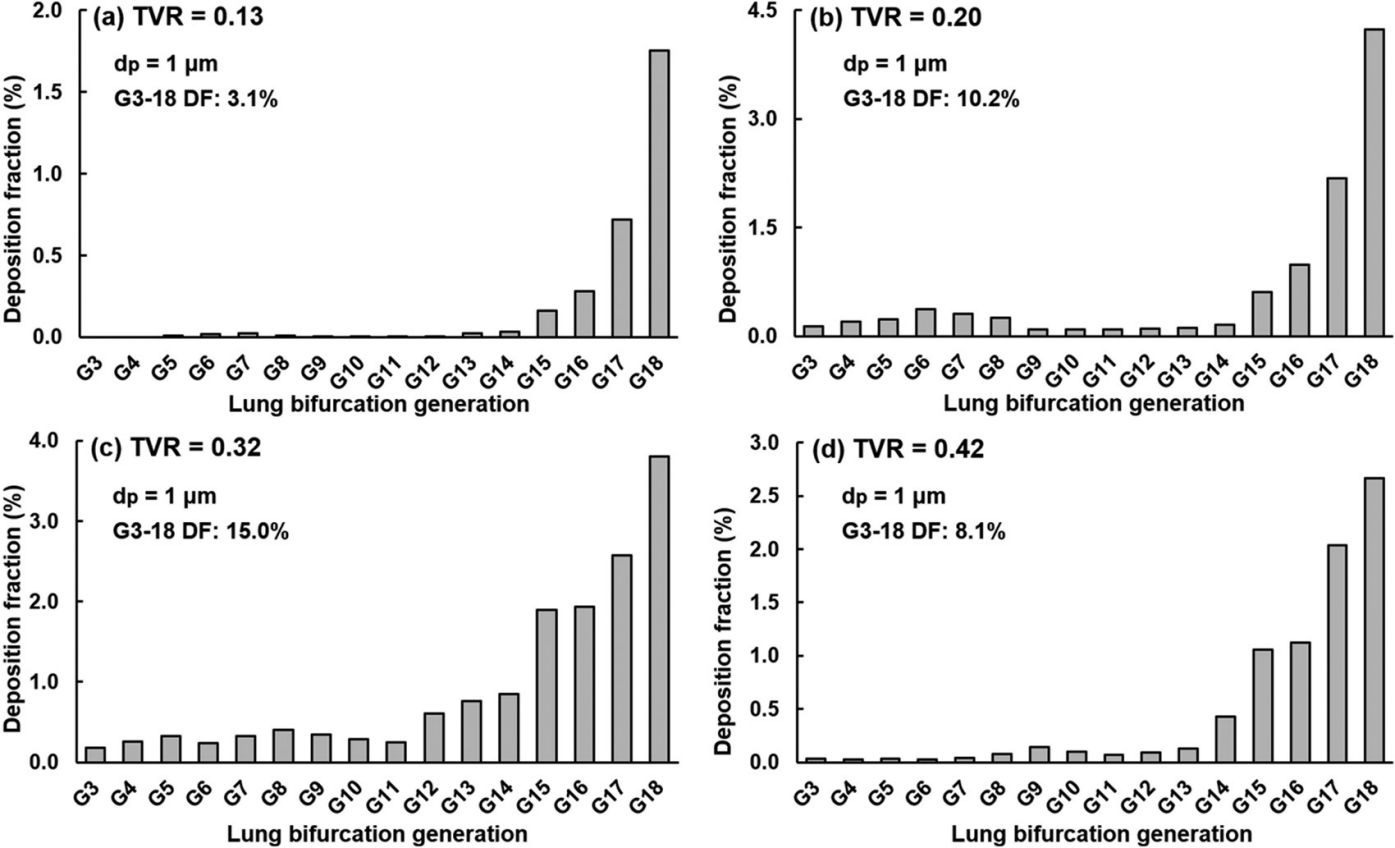
Deposition fraction of 1-*μ*m droplets from the alveoli in the lung branches G3–G18 at different cough depths: (a) TVR = 0.13, (b) 0.20, (c) 0.32, and (d) 0.42.

Deposition on the single-path lung branches at different generations (G3–G18) is shown in Fig. 11 for 1-*μ*m droplets. Deposition of droplets other than 1 *μ*m exhibits similar profiles and, thus, is not shown. Droplets from the alveoli predominately deposit in the respiratory bronchioles G15–G18, while few droplets deposit in the bronchioles beyond G15 [Figs. 11(a)–11(d)].

In contrast to the large (i.e., one order of magnitude, Figs. 7 and 8) variance in the droplet exhalation fractions, the DF variance in the lung branches is much smaller, as evidenced by an identical DF range for all cough depths considered (i.e., 0%–5%, Fig. 11). The cumulative DF in the G3–G18 branches ranges from 3.1% at TVR = 0.13 to 15.0% at TVR = 0.32. During a soft cough (TVR = 0.13), very few droplets deposit in the bronchioles beyond G15 and no droplet deposit in G3–4 [Fig. 11(a)]. A closer look at the DFs in G3–G14 [inset in Fig. 11(a)] reveals a similar pattern of DF vs branch generation as those at the other three cough depths, even though the magnitude of the generation-wise DFs is much lower at TVR = 0.13. A bell-shaped DF profile is observed for G5–G11 for all cough depths considered, despite that the branch generation number with the peak DF slightly differs among coughs. It is reminded that the single-path lung branch geometry strictly followed Weibel's lung dimension,^27^ whose specific dimension may give rise to the bell-shaped DF profiles observed herein.

#### 3. Upper airway

**FIG. 12.**
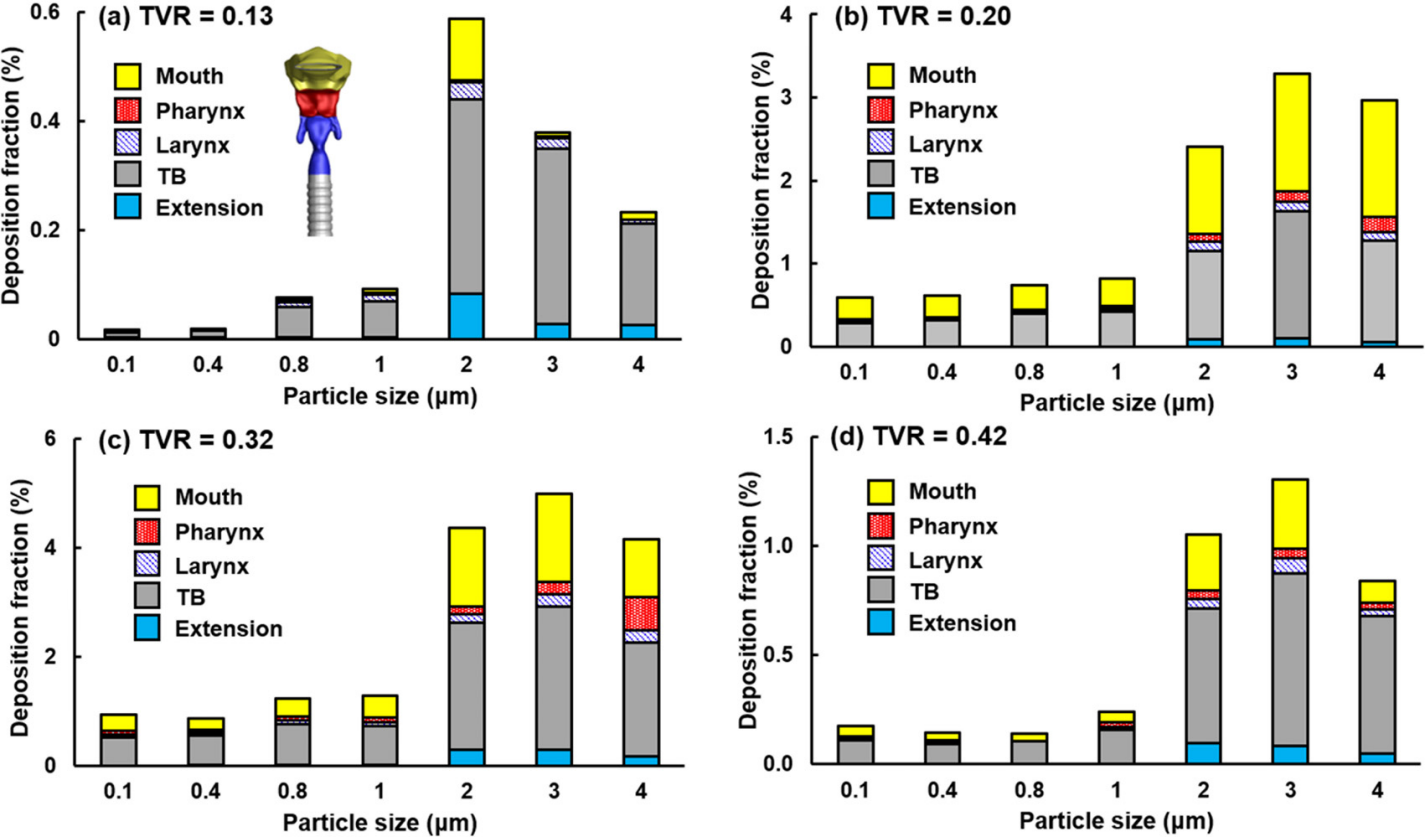
Deposition fractions of respiratory droplets (0.1–4 *μ*m) from the pulmonary alveoli in different regions of the extrathoracic airway at different cough depths: (a) TVR = 0.13, (b) 0.20, (c) 0.32, and (d) 0.42. Note that different ranges in the y-coordinate.

Figure 12 shows the regional deposition of pulmonary respiratory droplets in different regions of the extrathoracic airways for droplets ranging from 0.1 *μ*m to 4 *μ*m. Note that different ranges were used for the deposition rates (y-coordinate) at different cough depths. Again, much higher DFs were observed at TVR = 0.20 and 0.32 than at TVR = 0.13, which may result from the much larger number of particles escaping from the alveoli at TVR = 0.20 and 0.32 than at 0.13. In light of the particle size effect, more micrometer particles deposited in the upper airway than the submicrometer particles for all cough depths considered, which peaked at 2–3 *μ*m and declined at 4 *μ*m. For a given droplet size, the tracheobronchial (TB) region and mouth had most of the deposition, largely due to their larger surface areas in comparison to those of the pharynx and larynx.

## IV. DISCUSSION AND SUMMARY

In this study, we observed that among the respiratory aerosols ranging 0.1–4 *μ*m, 2 *μ*m droplets have the highest exhalation fraction from the pulmonary alveoli. This finding is consistent with Johnson *et al.*^11^ that the mean droplet diameter exhaled from deep lungs (B-mode) is 1.8 *μ*m during a cough. This observation can have important implications in understanding the role of coughs in transmitting COVID-19. Studies have shown that SARS-CoV-2 viruses first deposit in the human upper airway to cause infection of the nasal goblet secretory cells, and then spread to central and deep lungs.^59,60^ The final target is the alveolus,^61,62^ which is also one of the three confirmed binding sites for SARS-CoV-2 viruses with two necessary enzymes for cell invasion, ACE2 (angiotensin-converting enzyme 2) and TMPRSS2 (Type II transmembrane serine protease), coexist.^63,64^ The other two sites with these two enzymes coexisting are the nasal goblet cells and the ileal absorptive cells in the small intestine.^63^ As a result, respiratory droplets exhaled from the alveoli pose an especially high risk to the surrounding people; source controls such as wearing a mask to block the exhaled droplets from infected persons can be critical in reducing community transmissions.^65^ We now know that more than 50% of transmissions are from asymptomatic or pre-symptomatic infected persons;^66^ they are unaware of their infectiousness to others and do not feel obligated to wear a mask but will unintentionally emit a large amount of virus-laden droplets. Droplets of 2 *μ*m, once exhaled, can become submicrometer aerosols due to evaporation, which can stay airborne much longer than micrometer droplets and increase the chances of being inhaled by others.^67,68^ Wearing a mask by the infected host can block a large portion of these virus-laden droplets and slow down their exiting speeds to keep the droplets close by as well.^69^ Results of this study emphasize the need for facemasks and social distancing that can effectively block 2–3 *μ*m droplets to curb COVID-19 transmissions.

Previous studies have also shown that a surgical mask does not provide good protection to the wearer from airborne droplets smaller than 3 *μ*m.^70,71^ When referring to the protection efficiency of a mask, what we know about (like N95 being 95% effective) is the filtration efficiency of the mask material, not necessarily the efficiency it actually protects you from airborne viruses (i.e., personal protection efficiency).^72–74^ In a recent study,^75^ we demonstrated that wearing a low-filtration mask (say <25%) can inhale more particles smaller than 3 *μ*m than without a mask because the modified (lower) airflow favors the nasal inhalability of the 75%-particles that have escaped the mask filtration.^75^ Considering that the majority of the exhaled droplets from the deep lung are in the range of 2–3 *μ*m, these droplets can cast substantial risks to the surrounding person who even wears a mask. An alarming scenario is that mask-wearers do not realize that their old (low quality with low filtration efficiency) mask cannot protect themselves well from airborne PM_2.5_ (particulate materials 2.5 *μ*m and smaller), and with a false safety belief, ignore social distancing. It may cause them to inhale virus-laden droplets without realizing it if there happened to be one asymptomatic or pre-symptomatic infected person nearby. The results of this study not only promote mask-wearing as source control to block exhaled virus but also call for new filtration materials that are more effective in blocking PM_2.5_ for personal protection.

**FIG. 13.**
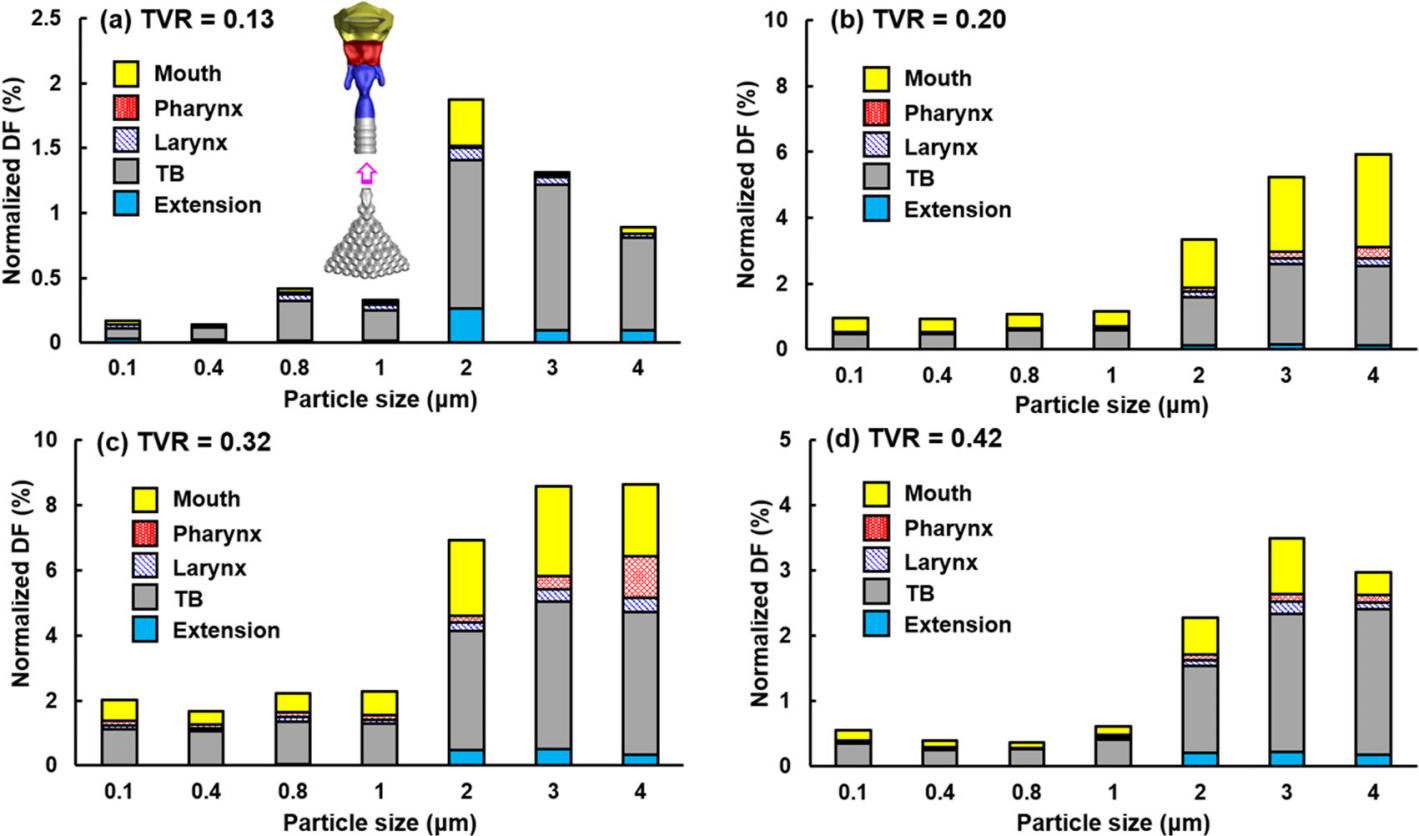
Normalized deposition fractions of respiratory droplets (0.1–4 *μ*m) by the number of droplets from the pulmonary alveoli: (a) TVR = 0.13, (b) 0.20, (c) 0.32, and (d) 0.42. Note that different ranges in the y-coordinate.

It is well expected that a soft cough will exhale a smaller number of droplets than a normal cough, as verified in this study. However, it is initially surprising that a hard or vigorous cough does not necessarily exhale linearly more droplets in comparison to a normal cough, or even exhale less. Results of this study show that normal and moderate coughs (TVR = 0.32 and 0.20) exhaled more droplets from the alveoli than a soft (TVR = 0.13) or vigorous (TVR = 0.42) cough does (Figs. 7 and 8). This nonlinear relationship between the cough depth and exhalation fraction of respiratory droplets can be attributed to several factors that concurrently determine the behavior and fate of these respiratory droplets. Note that an exhaled droplet needs to escape the filtration of the terminal alveoli, the single-path lung branches from G18 to G3, and the upper airway, including TB, larynx, pharynx, and the oral cavity. It needs to go through multiscale air passages (i.e., 0.2 mm in the alveolus to 40 mm in the mouth opening) and multiple flow regimes (laminar, transitional, and turbulent) with multiphysics deposition mechanisms (diffusion, sedimentation, inertial impaction, and transient effect). The lower alveolar retention rate at TVR = 0.20 and 0.32 [moderate and normal coughs, Figs. 10(b) and 10(c)] partially explains the higher exhalation fractions of respiratory droplets (Fig. 7) as well as the higher deposition fractions in the lung branches (Fig. 11) and upper airway (Fig. 12). To better understand the deposition mechanisms, the regional deposition fractions in the upper airways were normalized by the number of droplets escaping from the alveolar (i.e., dividing Fig. 12 by Fig. 10), thereby removing the confounding effects from the alveoli. As in Fig. 12, the resultant Fig. 13 fails to show any significant similarity between DFs of different cough depths or particle sizes, indicating a still complex flow and particle deposition mechanisms in the respiratory tract beyond the terminal alveolar. Indeed, high levels of turbulence and vorticity (Fig. 4) as well as droplet dispersion were spotted in the upper airway (Figs. 5 and 6). Even in the small airways G18–G13 with predominant laminar flows, droplet dispersion can occur due to flow transient effects (Fig. 6).

For a given mouth opening of 151.6 mm^2^, the mean flow velocity was 12.9, 19.8, 31.7, and 41.6 m/s at the peak of the coughs with TVR = 0.13, 0.20, 0.32, and 0.42, respectively. The mean velocities of the exhaled droplets were generally lower than the carrier flows, for instance, about 9 m/s for a soft cough (TVR = 0.13), 12–13 m/s for a moderate cough (TVR = 0.20), 18–20 m/s for a normal cough (TVR = 0.32), and 22–43 m/s for a vigorous cough (TVR = 0.42), as displayed in Fig. 9(d). The droplet exiting velocity became more sensitive to the droplet size when coughing harder. At TVR = 0.42, the exiting droplet speed was 43 m/s for 2–3 *μ*m and 22 to 30 m/s for submicrometer droplets [Fig. 9(d)]. Zhu *et al.*^15^ measured the speeds of cough droplets using particle image velocimetry (PIV) and reported the peak velocity varying from 6 to 22 m/s, with a mean of 11.2 m/s. Similarly, Chao *et al.*^76^ reported the peak cough velocities were 13.2 m/s and 10.2 m/s for males and females, respectively. A wider range of cough velocities (1.5–28.8 m/s) was reported by Tang *et al.*,^77–79^ who used a Schlieren optical system to visualize/quantify coughs. The droplet exiting speeds predicted in this study were overall higher than those measured by PIV or Schlieren systems (i.e., 18–20 m/s predicted vs 11–13 m/s measured for a normal cough). Two factors may contribute to this difference: the cross-sectional area of the mouth opening and the site of the velocity. A slightly wider opening of the mouth will lead to a lower exiting speed. More possibly, the lower measured speeds came from the quick loss of airflow–aerosol momentum in the stagnant ambient air, while the velocity was calculated by dividing the stooping distance and traveling duration, thus namely half of the peak velocity at the mouth opening. Using high-speed video, Nishimura *et al.*^80^ observed a fast decrease in particle speeds at 0.05 s after a cough and subsequently diffuse in the environmental airflow.

The applicability of the results from this study could be limited by several assumptions, which include local dynamic wall motions, an idealized single-path lung structure, a single source of respiratory droplets, and no droplet interactions. First, only alveolar wall motions were considered, which followed the experimentally measured cough waveform^28^ and were the driving force that dispensed aerosols in and out of the alveoli.^81^ Rigid walls were assumed for all other regions, including the upper airway and the single-path lung structure (with synchronized, transient flow boundary conditions at each branch). Particularly, the lung branch motions, glottal aperture variation, morphology variation of the oral cavity, and shape/size variation of the mouth-opening (lip positions) during a cough were neglected due to either the lack of data or prohibitive computational resources incurred. Second, only one single-path lung branch model was considered, whereas there are millions of possible pathways. In this study, the single-path lung structure extended to the base of the left lower lobe and generally aligned with the gravity for a sitting or standing subject, which makes the current alveoli more susceptible to be infected by virus-laden debris falling from the upper respiratory tract. Cough airflow and aerosol dynamics may be different from alveoli in different lobes with different orientations. Third, only droplets generated in the deep lung were considered. As aforementioned, respiratory droplets can be internally generated at different sites of the airway passage, including the glottis (or vocal folds) and the lips, in addition to film bursting in expanding respiratory bronchioles (deep lung). Furthermore, large shear stress during a cough can break loose mucus of the respiratory epithelium and generate virus-laden aerosols. In this study, droplets generated at the glottis and lips were not considered, which had been found to have larger droplet sizes (3.8 *μ*m and 374 *μ*m, respectively) than from the deep lung.^11^ The process of droplet formation in the peripheral airways was not considered in this study, as the understanding of aerosol generation in this region is still limited,^18–20^ and it is not the major objective of this study. Instead, aerosol droplets were assumed to stochastically fill the alveolar airspaces before coughing, and the range of the droplet size followed the measurements of Johnson *et al.*^11^ and Bake *et al.*^18^ Respiratory aerosol generation can be a highly complex process governed by multiple hydrodynamic stabilities, as demonstrated by Vadivukkarasan *et al.*, who experimentally studied the breakup morphology of an expelled respiratory fluid.^82^ Mechanisms of endogenous aerosol generation include reopening of closed airways and shear-induced destabilization of air–liquid interface.^18^ Specifically, small peripheral bronchioles can narrow or close after a deep exhalation; the reopening process at the start of the inhalation produces droplets. Fourth, droplet interactions, such as evaporation, collision, and associated breakup and aggregation, which have been demonstrated to exert varying levels of impact on respiratory droplet dynamics,^83^ were neglected. Considering that the temperature is constant and the relative humidity is close to saturation (as reported in Rouadi *et al.*^84^ and Keck *et al.*^85^), the evaporation/hygroscopic effect on the droplets should be insignificant inside the respiratory tract.^86^ The fate of exhaled droplets was not considered. Using a Schlieren imaging system, Simha and Rao demonstrated that the velocity of the cough cloud decays exponentially with distance, and the propagation is governed by expiratory vortex rings.^87^ Bhardwaj and Agrawal studied the drying time of exhaled droplets on a partially wetted surface and mask surfaces and correlated the chance of virus survival to influencing factors such as droplet volume, contact angle, ambient temperature, and humidity.^88–90^ The number of droplets was estimated to be 974–3000 per cough, with droplet concentrations ranging from 2.4 to 5.2 cm^−3^.^44,91,92^ As a result, the assumption of a dilute concentration should be valid and lends support to the negligence of droplet collisions.^93,94^ Also, particle size distribution,^95,96^ electrostatic charge,^97,98^ and evaporation and transport in the ambient air^67,99^ were not considered. Nevertheless, fundamental features of cough flow and droplet dynamics were captured and systemically investigated in this study, which includes a whole respiratory tract model with physiologically realistic cough kinematics and mouth opening. With the aforementioned simplifications, the evolution of a cough flow, as well as respiratory droplet behavior and fate, could be systematically examined by neglecting secondary, compounding factors.

In summary, a whole respiratory tract model was developed that extended from the mouth opening to the terminal alveoli following a single-path lung branch geometry. The shape and size of the mouth opening followed the image captured by a high-speed camera during the cough.^29^ Experimentally measured cough waveform and flow rate^28^ were used to control the contracting motions of the alveolar geometry that comprised 496 alveoli and 62 alveolar ducts.^40^ Synchronized flow boundary conditions at each branch with generation-specific flow rates altogether match the experimentally measured, transient cough flow rate. Cough airflow and droplet dynamics were simulated at four cough depths for respiratory droplets of 0.1–4 *μ*m. Specific findings of this study include:

1. For respiratory droplets of 0.1–4 *μ*m, the highest exhalation fraction (∼1.5%) from the alveoli occurs for 2 *μ*m droplets during a cough regardless of the cough depth.
2. A nonlinear relationship was observed between the droplet exhalation fraction and cough depth. While a soft cough (TVR = 0.13) exhaled fewer droplets than a normal one (TVR = 0.32), a vigorous cough (TVR = 0.42) also exhaled fewer droplets than a normal or moderate cough.
3. Droplets transport and deposition from the alveoli to mouth opening during a cough are featured by multiscale airway passages, multiregime flows, multiphysics deposition mechanisms, as well as drastic transient effects.
4. The droplet exiting speeds primarily depend on the cough depth. The mean velocity of the exhaled droplets is 18–20 m/s during a normal cough (TVR = 0.32), and the highest velocity can reach 50 m/s for individual droplets.
5. The peak exhalation fraction of 2 *μ*m virus-laden droplets from alveoli makes it more challenging to achieve personal protection by wearing a mask. Facemasks that are more effective in blocking PM_2.5_ are needed both for source control from the infected and filtration protection for the surrounding person.

## SUPPLEMENTARY MATERIAL

See the supplementary material for an animation of droplet motions during the cough was provided.

## Data Availability

The data used to support the findings of this study are available from the corresponding author upon reasonable request.
